# Diagnostic Applications of Ultrasound Imaging in Dental Implantology: A Systematic Review

**DOI:** 10.3390/jcm14228239

**Published:** 2025-11-20

**Authors:** Carlo Barausse, Subhi Tayeb, Martina Sansavini, Gerardo Pellegrino, Pietro Felice

**Affiliations:** 1 Oral Surgery Unit, Department of Biomedical and Neuromotor Sciences (DIBINEM), University of Bologna, 40125 Bologna, Italy; subhi.tayeb@unibo.it (S.T.); martina.sansavini@studio.unibo.it (M.S.); gerardo.pellegrino2@unibo.it (G.P.); pietro.felice@unibo.it (P.F.); 2 Department of Health Sciences, Magna Graecia University of Catanzaro, 88100 Catanzaro, Italy

**Keywords:** ultrasonography, ultrasound imaging, dental implants, systematic review, clinical studies, diagnosis

## Abstract

**Background/Objectives**: Conventional radiographic methods, although considered the gold standard for dental implantology, are not exempt from certain limitations, including their two-dimensional nature, the exposure to ionizing radiation and the inability to assess soft tissues. Ultrasonography (US) has recently emerged as a promising diagnostic tool due to its non-invasive and radiation-free properties. This systematic review aimed to evaluate the clinical applications of ultrasonography in implant dentistry, focusing on both preoperative planning and postoperative monitoring and to compare its diagnostic performance with conventional imaging modalities. **Methods**: A comprehensive search was performed in PubMed, Scopus, Web of Science, and Cochrane databases (2005–2025) to identify clinical studies evaluating the diagnostic applications of ultrasonography in implant dentistry. The review included randomized controlled trials, diagnostic accuracy studies, case series and case reports. Risk of bias was assessed using the RoB-2 tool for RCTs, QUADAS-2 for diagnostic studies and the JBI checklist for case reports and series. **Results**: 17 eligible studies were included in this review, comprising 4 RCTs, 10 diagnostic accuracy studies, 2 case report and 1 case series, for a total of 371 patients evaluated. Ultrasonography proved effective in the preoperative setting for evaluating mucosal thickness, keratinized mucosa, tissue phenotype, ridge width and bone morphology, often showing high agreement with CBCT and clinical measurements. In the postoperative phase, US enabled monitoring of soft tissue healing, vascular perfusion, graft maturation and detection of peri-implant pathology, with some studies showing correlations between early ultrasonographic findings and long-term clinical outcomes. Comparative studies revealed strong concordance with CBCT (mean deviations < 0.5–1 mm) and superior performance in both soft tissue visualization and cases affected by radiographic artifacts. **Conclusions**: Ultrasonography represents a promising adjunctive tool in implant dentistry, capable of complementing or, in selected scenarios, replacing conventional radiographic methods. While current evidence highlights its diagnostic potential across different stages of implant therapy, further standardized, large-scale clinical studies are required before routine integration into daily practice.

## 1. Introduction

Dental implants have become a widely accepted and increasingly utilized solution for the rehabilitation of partial or complete edentulism [1]. It is estimated that millions of implants are placed annually worldwide and this number is expected to rise as the global population ages and patient demand for fixed prosthetic solutions grows [2].

However, implant success is not solely determined by osseointegration; the long-term maintenance of healthy peri-implant tissues, including both soft tissue and underlying bone, plays a crucial role in ensuring clinical longevity [3,4]. Early detection of complications such as peri-implant mucositis, peri-implantitis and peri-implant bone defects is therefore essential for timely intervention and prevention of implant failure [5,6,7]. In this context, imaging modalities serve as pivotal diagnostic and monitoring tools during both pre-operative and post-operative phases, enabling comprehensive evaluation of both soft tissue health and peri-implant bone integrity [8]. Traditionally, intraoral periapical radiographs and orthopantomogram (OPG) have been the most frequently employed techniques to assess peri-implant bone levels [9]. However, both are two-dimensional and involve ionizing radiation, limiting their ability to evaluate soft tissues and subtle early changes [10]. Cone-beam computed tomography (CBCT) offers higher resolution and three-dimensional reconstructions, but its use remains limited due to radiation exposure and cost, especially for routine follow-up [11,12]. Ultrasound imaging (US), by contrast, is emerging as a promising diagnostic tool in implantology thanks to its ability to visualize peri-implant soft tissues in real time, without radiation and at relatively low cost [13,14,15,16,17]. Initially developed for medical applications, intraoral ultrasonography has evolved to include high-frequency probes capable of capturing detailed anatomical information within the oral cavity [18,19]. Its non-invasive nature, portability and increasing availability have fueled interest in its application not only for soft tissue monitoring but also for the evaluation of peri-implant bone defects and inflammatory changes [20,21,22]. Despite this potential, clinical integration of ultrasonography in implant dentistry remains limited. Several studies have explored its accuracy, reliability and comparability with conventional radiographic methods for assessing mucosal thickness, peri-implant bone defects, vascular perfusion and soft tissue elasticity [23,24]. However, heterogeneity in study design, populations, probe frequencies and imaging protocols makes it difficult to draw generalizable conclusions. Moreover, technical challenges such as probe positioning, image standardization and reproducibility across time points still need to be addressed [25]. The aim of this systematic review is therefore to investigate the different clinical applications of ultrasonography in implant dentistry [26,27,28,29]. In particular, it will analyze its use in the pre-operative phase (for surgical planning, evaluation of bone topography and mucosal thickness) as well as in the post-operative phase (for monitoring soft tissue thickness, vascular perfusion and peri-implant tissue healing) [30,31,32,33,34,35,36,37,38,39,40,41,42,43,44,45,46]. Despite the existence of previous systematic reviews on ultrasound applications in dentistry, most included preclinical or experimental studies and primarily focused on feasibility or imaging protocol optimization. The present review addresses this gap by exclusively analyzing human clinical studies, thereby providing a clinically relevant synthesis of ultrasonography’s diagnostic accuracy, reproducibility and practical applicability in both preoperative and postoperative phases of implant therapy. Furthermore, by integrating risk-of-bias assessment tools specific to each study design (RoB-2, QUADAS-2, and JBI), this review offers a structured and critical appraisal of the available clinical evidence, highlighting the translational potential of ultrasonography in daily implant practice. By addressing both diagnostic and follow-up perspectives, this review seeks to clarify whether ultrasonography can evolve into a fundamental tool for the implantologist, complementary or even alternative to conventional radiographic methods.

## 2. Materials and Methods

This systematic review aimed to critically evaluate the clinical applications of ultrasonography in the field of dental implantology.

The focused research question was formulated according to the PICO framework as follows:

**Population (P)**: patients receiving dental implants;
**Intervention (I)**: diagnostic use of ultrasonography for the assessment of peri-implant tissues;
**Comparison (C)**: conventional imaging modalities such as radiography or CBCT;
**Outcome (O)**: diagnostic accuracy, clinical applicability, and usefulness of ultrasonography in preoperative planning and postoperative monitoring.

The included studies assessed the use of ultrasound imaging in both preoperative and postoperative contexts, focusing on the evaluation of peri-implant soft and hard tissues, detection of peri-implant diseases, measurement of mucosal thickness and monitoring of bone remodeling over time. Particular attention was given to the diagnostic accuracy of ultrasonography compared to conventional radiographic techniques such as periapical X-rays and panoramic radiographs. Only clinical studies were included in order to assess the real-world applicability of ultrasound imaging on human patients. Although this review follows the PRISMA 2020 framework (Preferred Reporting Items for Systematic Reviews and Meta-Analyses) for systematic reviews [47] and was prospectively registered in PROSPERO (CRD420251118108), its exploratory and descriptive intent, driven by the heterogeneity of available clinical studies, also aligns with the methodological features of a scoping review. The review was conducted in accordance with Cochrane Collaboration methodological standards for systematic reviews of diagnostic studies, complemented by JBI guidance for integrating multiple study designs.

### 2.1. Search Strategy

A comprehensive literature search was conducted across PubMed, Scopus, Cochrane and Web of Science databases between 28 July and 1 August 2025. Search strings were adapted to each database while maintaining semantic consistency. A last comprehensive search was conducted on 3 August 2025 to perform a final screening of eligible articles across all databases.

The complete search strings and database-specific filters for all databases (PubMed, Scopus, Web of Science and Cochrane) are provided in Supplementary File S1.

Reference lists of selected articles were manually screened to identify additional relevant studies.

### 2.2. Inclusion and Exclusion Criteria

Studies were considered eligible for inclusion if they were published between 2005 and 2025 and focused on the application of ultrasonography for the assessment of peri-implant hard and soft tissues in human patients. Only clinical studies involving the use of ultrasound imaging for diagnostic purposes in implant dentistry were included. Studies had to evaluate the use of ultrasound in the preoperative phase (e.g., planning, tissue characterization) and/or postoperative phase (e.g., monitoring healing, detecting complications). To ensure clinical relevance, only studies assessing ultrasound use on patients receiving dental implants were included. Studies investigating ultrasound applications unrelated to implantology or those using ultrasound solely for therapeutic purposes were excluded. Additionally, only articles published in English with full-text availability were considered to guarantee data accessibility and quality. Exclusion criteria encompassed in vitro, animal and cadaveric studies. Reviews, editorials, conference abstracts without full text and studies focusing exclusively on non-diagnostic ultrasound technologies were also excluded. Finally, articles published in languages other than English or not available in full text were not included to ensure comprehensive data extraction and analysis.

### 2.3. Article Selection Process

**Figure 1:**
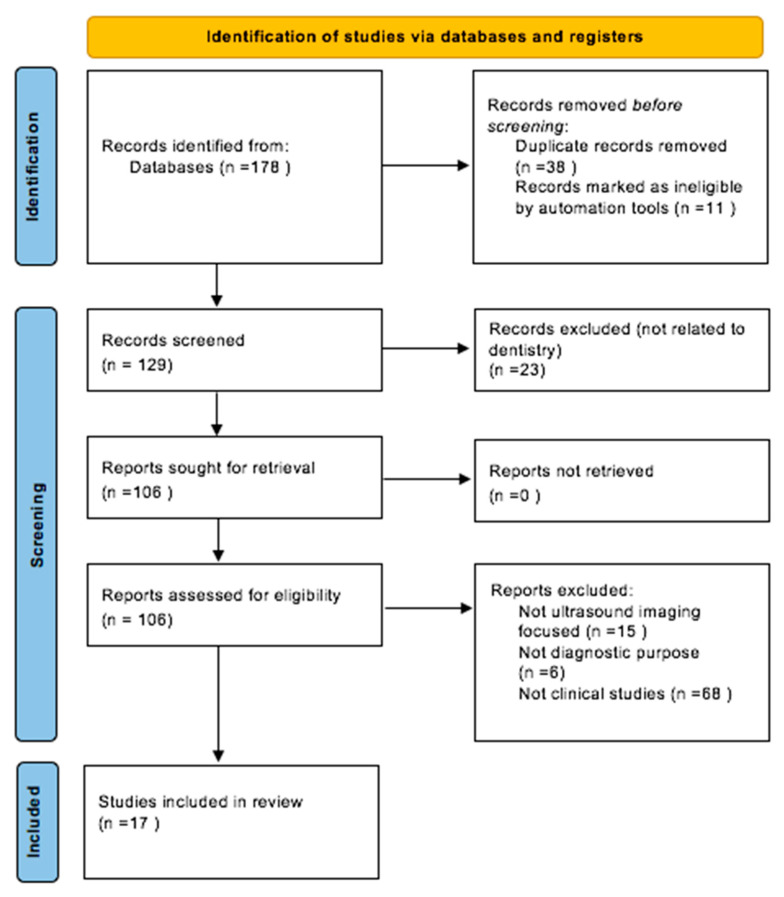
This diagram illustrates the systematic process of identifying, screening and selecting studies for inclusion in the systematic review.

The initial search identified 178 records among all databases. After applying filters for publication language (English) and publication date (2005–2025), 11 records were excluded, resulting in 167 records: 60 from PubMed, 73 from Scopus, 23 from Web of Science and 11 from Cochrane. Following the removal of 38 duplicates, 129 studies were screened based on titles and abstracts. Of these, 23 records were excluded for being unrelated to dentistry implantology. A total of 106 full-text articles were sought for retrieval. Of these, 89 were excluded for the following reasons: 15 did not focus on ultrasound imaging, 6 used ultrasound for therapeutic rather than diagnostic purposes and 68 were non-clinical studies. 17 studies met all inclusion criteria and were included in this systematic review. The selection process was conducted independently by two expert reviewers, with disagreements resolved through discussion with a third reviewer. The full selection process is summarized in the PRISMA flow diagram (Figure 1). The full list of excluded articles are provided in Supplementary File S2.

### 2.4. Data Extraction and Quality Assessment

Two independent reviewers performed data extraction using a standardized Excel spreadsheet.

The following information was collected from each included study: first author, year of publication, study design, number of patients, type of ultrasonography, application of the ultrasonography (preoperative and/or postoperative), surgical procedure, evaluated outcomes (mucosal thickness, crestal bone level, implant position, etc.), follow up and comparison with other diagnostic methods.

Any discrepancies between the two reviewers regarding study selection or risk of bias assessment were resolved through discussion with a third reviewer.

**Figure 2:**
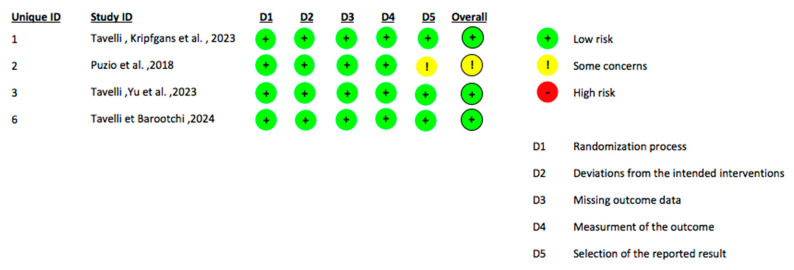
This table presents the assessment of the risk of bias for each included RCT, evaluated across the five domains of the RoB 2 tool [30,31,32,35].

**Figure 3:**
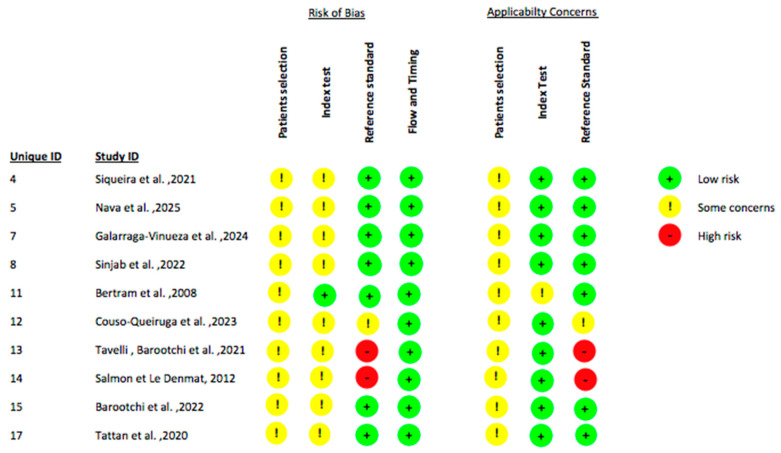
This table presents the assessment of the risk of bias for each included diagnostic accuracy study, evaluated across the four risk of bias domains and the three applicability domains of the QUADAS-2 tool [33,34,36,37,40,41,42,43,44,46].

**Figure 4:**
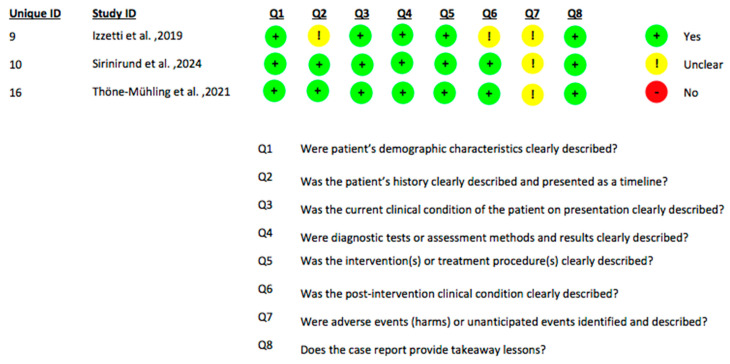
This table presents the quality appraisal for each included case report or case series, evaluated with the JBI Critical Appraisal Checklist [38,39,45].

Considering that this field of investigation is still in an early stage with a limited number of available clinical studies, the present systematic review incorporated studies involving diverse methodological designs, including randomized controlled trials (RCTs), observational diagnostic accuracy studies and case reports/series. Accordingly, risk of bias assessments were conducted employing tools specifically validated for each study design. The RoB 2 tool was utilized to appraise the risk of bias in randomized controlled trials (Figure 2) [48]. Observational studies with a diagnostic focus were evaluated using the QUADAS-2 tool to systematically assess methodological quality and bias risk related to diagnostic accuracy (Figure 3) [49]. For case reports and case series, the Joanna Briggs Institute (JBI) critical appraisal checklist was applied to evaluate study rigor and relevance (Figure 4). This stratified approach ensured an appropriate and robust critical appraisal tailored to the heterogeneity of included evidence. Regarding randomized trials, the main sources of bias were related to randomization procedures and lack of blinding of outcome assessment, whereas participant selection and data completeness were generally adequate. Diagnostic accuracy studies showed “some concerns” primarily due to variability in the reference standards used and the absence of predefined diagnostic thresholds. Case reports and case series were generally rated as low to moderate risk of bias, mainly due to limited sample size and incomplete reporting of patient selection criteria.

## 3. Results

This systematic review included seventeen clinical studies involving a total of 371 patients. No adverse clinical events or complications were reported, confirming the safety of this diagnostic method. Overall, the review included 4 RCTs, 10 diagnostic accuracy studies, 2 case reports and 1 case series. The analysis focused on three main aspects: pre-operative applications of ultrasonography (planning and baseline assessment), post-operative outcomes (monitoring of peri-implant tissues), and its diagnostic performance compared with established gold-standard methods.

The initial search identified 178 records among all databases. After applying filters for publication language (English) and publication date (2005–2025), 11 records were excluded, resulting in 167 records: 60 from PubMed, 73 from Scopus, 23 from Web of Science and 11 from Cochrane. Following the removal of 38 duplicates, 129 studies were screened based on titles and abstracts. Of these, 23 records were excluded for being unrelated to dentistry implantology. A total of 106 full-text articles were sought for retrieval. Of these, 89 were excluded for the following reasons: 15 did not focus on ultrasound imaging, 6 used ultrasound for therapeutic rather than diagnostic purposes and 68 were non-clinical studies. In Table 1 we summarize the findings of each study.

### 3.1. Pre-Operative Diagnostic Applications

#### 3.1.1. Soft Tissue Assessment and Phenotype Evaluation

Preoperatively, ultrasonography was employed across the included studies to provide a comprehensive evaluation of peri-implant tissues, both hard and soft, prior to surgical intervention. Several investigations showed its reliability in assessing soft tissue thickness and phenotype. Puzio et al. (2018) [31] showed that US could objectively differentiate thin from thick biotypes, with greater accuracy than clinical inspection alone, particularly in identifying thin tissues. Similarly, Tavelli, Yu et al. (2023) [32] confirmed that high-frequency US (HFUS) enabled a precise delineation of keratinized versus non-keratinized mucosa, providing linear and surface-based measurements of keratinized mucosa width that were comparable or superior to periodontal probing. Moreover, Tavelli & Barootchi (2024) [35] extended the scope of preoperative US to include strain elastography, highlighting that peri-implant mucosa was less elastic than gingiva around teeth, with elasticity values strongly correlated with keratinized mucosa width and mucosal thickness.

#### 3.1.2. Bone and Ridge Morphology Assessment

Beyond soft tissues, multiple studies validated the role of ultrasonography in the assessment of peri-implant bone levels and ridge morphology. Siqueira et al. (2021) [33] reported that US reliably measured facial bone level and cortical bone thickness, showing excellent correlation with intraoperative gold standards and CBCT and outperforming CBCT in cases of dehiscence or fenestration where radiographic artifacts occurred. Bertram et al. (2008) [40] validated these findings, showing that B-mode US could detect marginal bone loss with acceptable accuracy compared to surgical measurements, especially in cases of moderate bone defects. Tattan et al. (2020) [46] further showed that US could reproducibly quantify crestal bone level together with papilla height, midfacial soft tissue thickness and edentulous site morphology, with high agreement to CBCT and direct surgical measurements. Likewise, Sinjab et al. (2022) [37] validated US for edentulous ridge width and crest surface quality assessment, reporting excellent correlation with CBCT and proposing a clinically applicable classification of crest morphology.

#### 3.1.3. Vascular Perfusion and Soft Tissue Perfusion Mapping

Ultrasonography was also shown to provide valuable baseline information on vascular perfusion prior to grafting procedures. Tavelli, Kripfgans et al. (2025) [30] confirmed the hypothesis that Doppler ultrasonography could quantify perfusion at implant sites before soft tissue augmentation, establishing reproducible vascular parameters across treatment groups. Similarly, Tavelli, Barootchi et al. (2021) [42] applied Doppler US preoperatively to assess mucosal recession depth, peri-implant soft tissue thickness and palatal donor site characteristics, confirming its feasibility for vascular baseline analysis.

#### 3.1.4. Surgical and Prosthetic Planning

In addition to diagnostic applications, ultrasonography has been investigated as a tool for surgical and prosthetic planning. Nava et al. (2025) [34] combined US with intraoral scanning to achieve prosthetic-driven implant planning without radiation exposure, reporting clinically acceptable deviations between US-based planning and both CBCT planning and actual implant placement. Finally, Sirinirund et al. (2024) [39] confirmed that US could identify not only ridge width and soft tissue thickness but also adjacent anatomical structures such as frenula and muscles, thereby providing crucial guidance for flap design and regenerative procedures.

#### 3.1.5. Summary

Overall, the preoperative evidence consistently supports ultrasonography as a non-invasive, radiation-free, and reproducible tool capable of accurately characterizing both soft and hard peri-implant tissues. It reliably measures mucosal thickness, keratinized mucosa width, ridge width, and bone levels, showing strong agreement with CBCT and intraoperative findings. Moreover, its ability to visualize vascular perfusion and soft tissue elasticity offers unique diagnostic insights not achievable with conventional radiography, highlighting its potential as a comprehensive imaging modality for surgical and prosthetic planning.

### 3.2. Post-Operative and Follow-Up Applications

#### 3.2.1. Monitoring of Soft Tissue Healing and Thickness Changes

Postoperatively, ultrasonography has been widely employed to monitor both structural and functional changes in peri-implant tissues over time. A consistent body of evidence supported its value in tracking soft tissue healing and augmentation outcomes. Puzio et al. (2018) [31] showed that US accurately quantified gains in mucosal thickness following soft tissue grafting, detecting differences between CTG (Connective Tissue Graft) and xenogeneic matrices at 3 months and documenting partial volumetric loss at longer follow-ups, while still maintaining clinically relevant improvements compared to baseline. Similarly, Tavelli, Yu et al. (2023) [32] confirmed that high-frequency US provided detailed measurements of keratinized mucosa width after augmentation procedures, with surface-based analysis offering a more precise three-dimensional understanding than linear or clinical probing. Moreover, Tavelli & Barootchi (2024) [35] employed elastography to assess peri-implant mucosal stiffness after CTG grafting, showing a progressive increase in rigidity between 6 and 12 months, indicative of tissue maturation and integration.

#### 3.2.2. Assessment of Vascular Perfusion and Tissue Remodeling

Beyond structural assessments, several studies applied Doppler US to evaluate vascular perfusion dynamics during healing. Tavelli, Kripfgans et al. (2025) [30] proved distinct revascularization patterns between CAF (Coronally Advanced Flap) and TUN (Tunnel) techniques, with early increases in perfusion correlating with long-term mucosal thickness and defect coverage. Likewise, Tavelli, Barootchi et al. (2021) [42] reported transient peaks in perfusion at 1 week and 1 month after grafting, followed by normalization at 6–12 months, reflecting the transition from inflammatory healing to tissue stability. Sirinirund et al. (2024) [39] confirmed these findings, showing temporary increases in soft tissue perfusion and dimensional changes in the ridge during healing, with US findings corroborated by surgical re-entry.

#### 3.2.3. Detection of Peri-Implant Complications

A growing number of studies have further highlighted the role of US in monitoring peri-implant tissue conditions following implant placement, particularly in the diagnosis and follow-up of peri-implant diseases. Barootchi et al. (2022) [44] quantified perfusion patterns in sites with health, mucositis and peri-implantitis, showing significantly higher vascular indices in diseased sites and excellent diagnostic discrimination. Galarraga-Vinueza et al. (2024) [36] identified a hypoechoic supracrestal area as a hallmark of peri-implantitis, perfectly distinguishing diseased from healthy tissues, with strong associations to bleeding, suppuration and bone loss. Izzetti et al. (2019) [38] employed US to characterize postoperative complications, identifying inflammatory lesions, graft remnants and periosteal reactions not appreciable by radiography and integrating this information with CBCT for comprehensive diagnosis.

#### 3.2.4. Long-Term Monitoring and Maintenance-Phase Evaluation

Finally, US has been successfully applied to long-term follow-up and cross-sectional evaluation of implants in function. Thöne-Mühling et al. (2021) [45] used high-resolution intraoral US to assess mucosal recession, keratinized tissue width and buccal bone loss in implants functioning for over a decade, reporting good correlation with radiographic and surgical findings and proving feasibility for real-time perfusion imaging. Couso-Queiruga et al. (2023) [41] further confirmed its reproducibility in measuring peri-implant mucosal thickness across 100 healthy implants, showing excellent agreement with CBCT-derived STL models. Salmon & Le Denmat (2012) [43] also underscored the practical feasibility of intraoral US for routine postoperative monitoring, emphasizing its rapid, non-invasive and well-tolerated nature.

#### 3.2.5. Summary

Collectively, postoperative evidence indicates that ultrasonography enables precise and dynamic monitoring of peri-implant soft tissues, vascular perfusion, and bone morphology over time. It effectively detects changes following grafting procedures and differentiates healthy from diseased peri-implant sites, confirming its role as a reliable adjunct for clinical follow-up and early complication detection.

### 3.3. US Outcomes and Comparison with Conventional Diagnostic Methods

#### 3.3.1. Comparison with CBCT and Radiography

When compared with conventional diagnostic approaches, ultrasonography has consistently shown high accuracy and reproducibility, while offering additional advantages such as the absence of ionizing radiation and the ability to visualize soft tissues in real time. Several studies directly compared US with cone-beam computed tomography (CBCT), reporting excellent correlations for the assessment of bone crest width, cortical thickness and facial bone level (Siqueira et al., 2021 [33]; Sinjab et al., 2022 [37]; Tattan et al., 2020 [46]). In Sinjab’s [37] study, the correlation between US and CBCT for ridge width was extremely high (r = 0.96–0.98), with mean differences ranging from only 0.048 mm at 1 mm from the crest to 0.18 mm at 3 mm and a 91% agreement in the qualitative assessment of the crestal surface. Similarly, Siqueira et al. [33] reported strong correlations between US and direct intraoperative measures (r^2^ = 0.95 for facial bone level) and CBCT (r^2^ = 0.85 for cortical thickness). Importantly, in cases with thin buccal bone, dehiscence or fenestration, US outperformed CBCT, which was often affected by beam hardening artifacts and failed to capture fine structural details. In the context of implant planning, Nava et al. (2025) [34] showed that US-guided protocols achieved deviations of 0.92 mm at the entry point, 1.41 mm at the apex and 5.27° of angular deviation compared with CBCT-based planning, while the deviations between US planning and the actual implant position remained within 1.16 mm (entry), 1.26 mm (apex) and 2.63° (angle). All implants planned with US were successfully placed within the bony envelope, highlighting the clinical applicability of this approach. Similarly, Couso-Queiruga et al. (2023) [41] reported good agreement between US- and CBCT-derived DICOM-STL measurements of mucosal thickness, with Intraclass Correlation Coefficients (ICC) around 0.81–0.82 and mean differences below 0.25 mm.

#### 3.3.2. Correlation with Intraoperative and Clinical Measurements

In relation to direct intra-surgical measurements, ultrasonography proved equally reliable. Both Bertram et al. (2008) [40], as well as Tattan et al. (2020) [46], showed that US measurements of peri-implant bone levels and soft tissue dimensions closely matched those obtained during flap surgery, with mean differences below 0.5 mm and ICC values up to 0.88, confirming its reproducibility. This highlights the potential of US to provide diagnostic accuracy similar to intraoperative evaluation, but in a non-invasive manner.

When compared with periodontal probing, US offered more objective and reproducible assessments. Puzio et al. (2018) [31] reported that US was more accurate in differentiating thin versus thick mucosal biotypes than clinical inspection or probing, while Tavelli, Yu et al. (2023) [32] displayed that HFUS could distinguish keratinized from non-keratinized mucosa with high precision, with surface-based measurements of keratinized mucosa width reaching 5.31 ± 1.19 mm compared to 3.98 ± 1.25 mm measured clinically, underscoring the improved spatial resolution of US.

#### 3.3.3. Doppler Ultrasonography for Perfusion and Inflammatory Assessment

Several studies also highlighted the added value of US in functional and perfusion analysis, which cannot be achieved with radiographs or clinical probing. Tavelli, Barootchi et al. (2021) [42] showed that Doppler US detected a peak increase in blood volume up to +199% at 1 week post-surgery, which progressively normalized below baseline at 6–12 months, paralleling tissue healing. Tavelli, Kripfgans et al., 2025 [30] found that sites treated with tunnel technique (TUN) plus CTG displayed significantly higher perfusion indices during the first month compared to CAF + CTG and early perfusion values correlated with greater mucosal thickening and coverage at 12 months. Moreover, Barootchi et al., 2022 [44] proved that perfusion values (Color Velocity and Color Power) were significantly elevated in peri-implantitis sites compared to healthy implants, correlating positively with probing depth, bleeding and suppuration. Galarraga-Vinueza et al., 2024 [36] identified a distinct hypoechoic supracrestal area (HSA) in 100% of peri-implantitis sites and in none of the healthy ones, with a mean vertical extension of 4.26 ± 1.48 mm and area ratio of 37.9% ± 14.8%. The diagnostic accuracy of HSA for distinguishing health from peri-implantitis reached an AUC of 1, indicating perfect discrimination.

In summary, ultrasonography provides diagnostic performance comparable to CBCT and direct surgical measurements for both bone and soft tissue evaluation, while surpassing conventional methods by offering dynamic, real-time, and radiation-free information. These findings support its clinical applicability as a complementary imaging modality in implant dentistry.

### 3.4. Safety and Adverse Events

No adverse events associated with the use of ultrasonography were reported in any of the included studies. The non-ionizing and non-invasive nature of the technique was consistently emphasized as a major advantage, particularly in scenarios requiring repeated follow-up examinations.

### 3.5. Limitations

The findings of this review indicate that ultrasonography holds a promising diagnostic role in implant dentistry, with relevant applications in both preoperative planning and postoperative monitoring of peri-implant tissues. Nonetheless, several methodological limitations should be considered when interpreting the current evidence. The included studies are heterogeneous in design, population characteristics, and ultrasound protocols, and only a limited number of randomized controlled trials are available. Differences in transducer frequency, imaging mode, acquisition settings and follow-up duration may affect spatial resolution, measurement reproducibility, and the comparability of outcomes across studies. Such variability precluded a quantitative meta-analysis, restricting the synthesis to a qualitative interpretation. The potential presence of publication bias should also be acknowledged. The predominance of positive findings and small sample sizes suggests that negative or inconclusive results may be underrepresented in the literature. Together with the variability in risk-of-bias ratings and incomplete methodological reporting, these factors limit the overall strength and generalizability of the available evidence. Collectively, the current data support the diagnostic potential of ultrasonography in implant dentistry, but the overall level of evidence remains moderate and exploratory. Further multicenter clinical trials using standardized imaging protocols, rigorous study designs and preregistered methodologies are needed to confirm diagnostic accuracy and establish clear guidelines for clinical implementation.

## 4. Discussion

The present systematic review aimed to critically evaluate the clinical applications of ultrasonography in implant dentistry, focusing on its diagnostic accuracy and utility in both pre-operative planning and post-operative monitoring. As the literature already suggests, ultrasonography has been increasingly investigated in the dentomaxillofacial region, with reviews emphasizing its diagnostic potential across a wide range of applications (Evirgen & Kamburoğlu, 2016 [17]; Reda et al., 2021 [16]). In implantology specifically, a previous systematic review summarized potential clinical indications of ultrasonography, including peri-implant soft tissue and bone assessment (Bhaskar et al., 2018 [14]). The present review builds upon this body of evidence by systematically analyzing human clinical studies, thereby providing a more practice-oriented evaluation of its diagnostic performance.

Preoperative applications revealed that ultrasonography is a reliable method for characterizing peri-implant soft tissues, including mucosal thickness, keratinized mucosa width and tissue phenotype (Puzio et al., 2018 [31]; Tavelli, Yu et al., 2023 [32]; Tavelli & Barootchi 2024 [35]; Sirinirund et al., 2024 [39]; Tattan et al., 2020 [46]). Several studies showed its accuracy in differentiating thin versus thick biotypes and in delineating the transition between keratinized and non-keratinized mucosa, offering measurements that were comparable or superior to clinical probing. Furthermore, US proved accurate for evaluating ridge width, crestal bone thickness and buccal bone level, showing excellent correlation with both CBCT and direct intra-surgical measurements (Siqueira et al., 2021 [33]; Sinjab et al., 2022 [37]; Salmon et al., 2012 [43]). Importantly, in scenarios with thin buccal plates or the presence of dehiscence and fenestration, ultrasonography outperformed CBCT, which was often limited by beam-hardening artifacts. These findings underscore its potential as a valuable diagnostic aid in implant planning (Nava et al., 2025 [34]), particularly when a radiation-free approach is desirable.

Postoperative monitoring further highlighted the added value of ultrasonography. High-frequency probes combined with Doppler modalities enabled the detection of soft tissue volumetric changes following grafting, as well as dynamic assessment of vascular perfusion and tissue elasticity over time (Tavelli, Kripfgans et al., 2025 [30]; Tavelli, Barootchi et al., 2021 [42]; Barootchi et al., 2022 [44]). Early increases in perfusion were shown to correlate with long-term stability of augmented tissues, while elastography confirmed progressive stiffening of grafted connective tissue during maturation. Beyond graft monitoring, US presented strong diagnostic potential in distinguishing healthy peri-implant tissues from sites affected by mucositis or peri-implantitis (Galarraga-Vinueza et al., 2024 [36]; Izzetti et al., 2019 [38]; Thöne-Mühling et al., 2021 [45]). Characteristic sonographic features, such as hypoechoic supracrestal areas and increased perfusion indices, were strongly associated with clinical and radiographic indicators of disease. Moreover, case reports and cross-sectional studies showed that ultrasonography can detect complications, soft tissue lesions, and marginal bone loss not readily visualized with conventional radiography (Izzetti et al., 2019 [38]; Bertram et al., 2008 [40]; Thöne-Mühling et al., 2021 [45]).

When compared with established diagnostic methods, ultrasonography consistently proved strong agreement with CBCT (Siqueira et al., 2021 [33]; Sinjab et al. [37]; Couso-Queiruga et al., 2023 [41]) and intraoperative measurements, while surpassing periodontal probing in objectivity and reproducibility. Unlike radiographic modalities, US provides additional functional insights into vascularity and tissue quality, which may prove clinically relevant for early detection of disease and evaluation of treatment response. At the same time, it should be emphasized that CBCT remains indispensable for complex three-dimensional evaluations, particularly in cases requiring detailed visualization of anatomical structures or advanced bone reconstruction. From a clinical perspective, the evidence suggests that ultrasonography can become a useful adjunct for implantologists, supporting both prosthetically driven planning and long-term follow-up of peri-implant tissues. Its ability to combine structural, functional and dynamic information in a non-invasive manner represents a unique advantage, already suggested by previous studies (Bhaskar et al., 2018 [14]; Chan et al., 2017 [15]).

However, current literature is limited by heterogeneity in study designs, small sample sizes, and variability in probe frequencies and imaging protocols. Most of the available evidence derives from pilot studies, case series, or cross-sectional designs, with relatively few randomized controlled trials. Another relevant limitation concerns the operator dependency and equipment variability inherent to ultrasonography. Image quality and diagnostic accuracy are strongly influenced by probe frequency, transducer design, and the clinician’s experience in maintaining consistent probe angulation and contact. This variability may partially explain differences in outcomes across studies and underlines the importance of training and standardization of scanning protocols. Future research should focus on well-designed longitudinal clinical trials to validate the diagnostic thresholds of ultrasonography, establish standardized imaging protocols and explore integration with digital workflows such as intraoral scanning and CAD/CAM planning. The development of user-friendly, high-resolution intraoral probes and automated image analysis software will also be critical to facilitate its clinical adoption.

An additional source of heterogeneity across studies concerns the variability of ultrasound devices and imaging protocols. Proprietary systems such as Pirop^®^ (for A-scan mucosal thickness measurement) and Mindray or Zonare platforms (for B-mode and Doppler analyses) differ in transducer frequency, beam geometry, and calibration settings. These factors directly influence spatial resolution, penetration depth, and quantitative accuracy, making cross-study synthesis more challenging. Moreover, the choice between A-scan and B-mode imaging entails different diagnostic capabilities (A-scan providing high precision for linear soft-tissue measurements, while B-mode allows for qualitative and spatial tissue evaluation). The lack of standardized reporting on device specifications and imaging settings across studies limits reproducibility and may partly explain variability in the reported outcomes. When interpreted collectively, these factors highlight that while the diagnostic potential of ultrasonography in implant dentistry is promising, the current evidence base should be considered moderate in quality and exploratory in nature. In conclusion, ultrasonography has revealed promising diagnostic performance for both preoperative evaluation and postoperative monitoring in implant dentistry. While it cannot yet replace radiographic methods, it provides complementary structural and functional information that may enhance clinical decision-making. With further refinement and validation, ultrasonography has the potential to become a fundamental tool in the daily practice of implantology.

## 5. Conclusions

Ultrasonography is emerging as a reliable, non-invasive and radiation-free imaging modality in implant dentistry. Evidence from the available clinical studies shows that US can provide accurate information in both preoperative phases and postoperative monitoring. Comparisons with conventional diagnostic tools such as CBCT, radiography and direct clinical measurements indicate that US offers comparable or superior diagnostic performance in specific contexts, particularly in soft tissue evaluation and in cases where radiographic artifacts limit reliability.

Despite these encouraging results, heterogeneity in study designs, probe frequencies, imaging protocols and limited sample sizes restricts the generalizability of findings. Further large-scale, standardized investigations are necessary to validate ultrasonography as a routine tool in implant dentistry. Nevertheless, current evidence supports its role as a valuable adjunct and potentially a fundamental instrument, for clinicians aiming to improve diagnostic accuracy and patient safety in both the planning and long-term maintenance of dental implants.

## Figures and Tables

**Table 1:** This table provides an overview of the studies selected for inclusion in the systematic review. It outlines key information for each study.

Title	Authors	Study Design	Number of Patients	Ultrasound Type	Ultrasound Application	Surgical Procedure Evaluated with the Ultrasound	Outcomes Assessed	Follow Up	Comparison with Other Diagnostic Methods
Doppler ultrasonographic evaluation of tissue revascularization following connective tissue graft at implant sites	Tavelli, Kripfgans et al., 2025 [30]	RCT	28	High-frequency ultrasound (HFUS) system with Colour Doppler Velocity (CDV) and Power Doppler Imaging (PDI)	Both pre-operatively and post-operatively	CTG for peri-implant soft tissue augmentation using CAF or TUN	(1) Changes in the tissue perfusion (2) Early revascularization of the CTG (3) Direction of perfusion	Preoperative 1 week 1 month 6 months 12 months	No comparison
Ultrasound assessment of soft tissue augmentation around implants in the aesthetic zone using a connective tissue graft and xenogeneic collagen matrix—1-year randomised follow-up	Puzio et al., 2018 [31]	RCT	57	Pirop^®^ ultrasonic device (Echoson, Puławy, Poland), A-scan probe, 20 MHz frequency, measurement range 0.25–6 mm, accuracy ±0.01 mm	Both pre-operatively and post-operatively	Soft tissue augmentation around implants in the aesthetic zone using either CTG or xenogeneic collagen matrix	(1) Thickness of keratinized tissue (TKT) at the cemento-enamel junction (CEJ) (2) Thickness of keratinized tissue (TKT) at the mucogingival junction (MGJ)	Preoperative 3 months 12 months	Direct periodontal probing with a manual probe
Keratinized mucosa width assessment at implant sites using high-frequency ultrasonography	Tavelli, Yu et al., 2023 [32]	RCT	28	Miniature HFUS transducer, 24 MHz, B-mode, high axial resolution (64 μm).	Both pre-operatively and post-operatively	CTG for peri-implant soft tissue augmentation using CAF or TUN	(1) Keratinized Mucosa width (KM) around dental implants (2) Mucosal Thickness (MT) around dental implants	Preoperative 12 months	Direct periodontal probing with a manual probe (PCP UNC 15)
Comprehensive peri-implant tissue evaluation with ultrasonography and cone-beam computed tomography: A pilot study	Siqueira et al., 2021 [33]	Retrospective pilot observational diagnostic accuracy study	4	High-resolution, 24 MHz, intraoral probe (ZS3, Mindray, Shenzhen, China) with B-mode and color-flow imaging	Pre-operatively	(1) Revision/implant removal surgery due to peri-implantitis (2) Second-stage uncovery surgery	(1) Crestal Bone Thickness (CBT) (2) Facial Bone Level (FBL) (3) Soft tissue characteristics (4) Blood flow within the soft tissue	No follow up. The evaluations were all performed at the time of the surgical procedure	(1) CBCT (2) Direct measurements (intraoperative photographs and a digital modeling)
Ultrasonography-Guided Dental Implant Surgery: A Feasibility Study	Nava et al., 2025 [34]	Prospective diagnostic feasibility study	9	S3 Mindray ultrasound + L30-8 linear probe, 24 MHz, 32.1 μm resolution	Both pre-operatively and post-operatively	Fully guided implant placement using a tooth-supported surgical guide	(1) Soft tissue profile deviation (2) Implant planning	No follow-up. Evaluations were performed pre-operatively for planning and immediately post-operatively to verify implant position	(1) CBCT-based implant planning (2) Intraoperative verification (actual implant positions measured during surgery were compared with the US-based plan)
Soft tissue elasticity at teeth and implant sites. A novel outcome measure of the soft tissue phenotype	Tavelli & Barootchi 2024 [35]	RCT	28	Ultrasound device: ZS3 (Zonare/Mindray, Mahwah, NJ, USA) Transducer: L30-8, 24 MHz (Zonare/Mindray, Mahwah, NJ, USA)	Both pre-operatively and post-operatively	CTG for peri-implant soft tissue augmentation using CAF or TUN	(1) Mucosal thickness (2) Gingival thickness (3) Buccal bone dehiscence (BBD) (4) Tissue elasticity/strain ratios (SR1, SR2, SR3)	Preoperative 6 months 12 months	Direct periodontal probing with a manual probe
Echo-intensity characterization at implant sites and novel diagnostic ultrasonographic markers for peri-implantitis	Galarraga-Vinueza et al., 2024 [36]	Diagnostic accuracy study	60	High-frequency ultrasound (HFUS) (The authors did not provide details regarding the brand and model of the ultrasound device employed)	Post-operatively	Implant placement and loading	(1) Mucosal thickness (2) Keratinized tissue width (3) Mucosal recession (4) Supracrestal tissue height (STH) (5) Soft tissue area (STA) (6) Hypoechoic supracrestal area (HSA) (7) Proportion HSA/STA (8) Buccal bone dehiscence (9) Crown angle (10) Greyscale texture outcomes	The ultrasound was used once to image implants that had already been in function for at least 1 year	(1) Direct periodontal probing (2) Periapical radiographs
Ultrasonographic Evaluation of Edentulous Crestal Bone Topography: A Proof-of-Principle Retrospective Study	Sinjab et al., 2022 [37]	Retrospective diagnostic accuracy study.	28	Ultrasound device: ZS3 (Mindray of North America, Mountain View, CA, USA) Prototype probe (L25–8, 24-MHz transmit frequency, Mindray, Shenzhen, China)	Pre-operatively	Healed edentulous sites scheduled for implant placement	(1) Bone ridge width (BRW) (2) Crestal bone surface quality (CBSQ)	No follow up. The evaluations were all performed before the surgical procedure	CBCT
Feasibility of a combination of intraoral UHFUS and CBCT in the study of peri-implantitis	Izzetti et al., 2019 [38]	Case report	1	Vevo MD system (Fujifilm VisualSonics, Toronto, Canada) 70 MHz probe (ultra-high-frequency ultrasonography, UHFUS)	Post-operatively	Implant placement followed by a Guided Bone Regeneration (GBR)procedure (membrane + allogeneic bone graft)	(1) Width of the lesion (2) Degree of soft tissue alteration (3) Vascularity of the swollen mucosa (4) Identification of hypoechoic periosteal alterations and hyperechoic graft remnants	No follow up. The evaluations were all performed immediately after the onset of postoperative complications	CBCT
Ridge augmentation planning, wound healing evaluation, and peri-implant tissue phenotype assessment with ultrasonography: A case report	Sirinirund et al., 2024 [39]	Case report	1	Ultrasound device: (ZS3, Mindray of North America, Mountain View, CA, USA) L30-8 probe	Both pre-operatively and post-operatively	Tooth extraction: GBR with allograft + resorbable membrane. Implant placement with simultaneous GBR. Second-stage implant surgery with free gingival graft and crown delivery.	(1) Soft tissue thickness (2) Crestal bone width (3) Muscle attachment location (4) Soft-hard tissue interface morphology (5) Tissue perfusion (fractional color density with color/power Doppler) (6) Peri-implant tissue phenotype at 1 year after implant loading	Baseline 1 month 2.5 month 5 month 12 months	No comparison
Sonography of peri-implant buccal bone defects in periodontitis patients: A pilot study	Bertram et al., 2008 [40]	Diagnostic accuracy pilot study	25	Sonoace Pico (Sonoace GmbH, Marl, Germany) Linear B-scan 12.5 MHz small-part transducer	Pre-operatively	Periodontal flap surgery for patients with residual deep pockets (>6 mm) and peri-implant buccal bone loss.	Vertical distance between the upper implant thread and the most apical level of marginal buccal bone (linear bone loss measurement)	No follow up. Evaluations were all performed before and at the time of the surgical procedure	Direct periodontal probing with a manual probe
Non-invasive assessment of peri-implant mucosal thickness: A cross-sectional study	Couso-Queiruga et al., 2023 [41]	Cross-sectional diagnostic accuracy study	50	Non-ionizing ultrasound biometer (PIROP, Echo-Son, Pulawy, Poland) Probe frequency 20 MHz	Post-operatively	Implants already placed and restored before the study (diagnostic evaluation only)	Facial mucosal thickness (FMT) measured 3 mm apical to the mid-facial mucosal margin of each implant.	The ultrasound was used once to image implants that had already been in function for at least 1 year	(1) CBCT (2) CBC+STL
Ultrasonographic tissue perfusion analysis at implant and palatal donor sites following soft tissue augmentation: A clinical pilot study	Tavelli, Barootchi et al., 2021 [42]	Prospective clinical pilot diagnostic study	5	Power Doppler ultrasonography with a high-frequency linear transducer (20 MHz, 35-mm footprint)	Both pre-operatively and post-operatively	CAF combined with a subepithelial CTG for treatment of peri-implant soft tissue dehiscence defects at the implant site	Vascularization/perfusion indices at implant and palatal donor sites	Baseline 1 week 2 weeks 6 weeks	No comparison
Intraoral ultrasonography: development of a specific high-frequency probe and clinical pilot study	Salmon & Le Denmat 2012 [43]	Prospective clinical pilot feasibility study	3	Custom-made intraoral high-frequency B-mode ultrasound probe, 25 MHz linear transducer for periodontal, peri-implant and soft tissue imaging	Post-operatively	Implants already placed and restored before the study (diagnostic evaluation only)	Visualization of periodontal, peri-implant and soft tissue structures	The ultrasound was used once to image implants that had already been in function	No comparison
Ultrasonographic Tissue Perfusion in Peri-implant Health and Disease	Barootchi et al., 2022 [44]	Cross-sectional diagnostic case–control study	21	Color flow and power Doppler ultrasonography, high-frequency linear transducer, used to quantify peri-implant tissue perfusion (color velocity, color power)	Post-operatively	Implants already placed and restored before the study (diagnostic evaluation only)	Peri-implant tissue perfusion	The ultrasound was used once to image implants that had already been in function for at least 3 years	(1) Direct periodontal probing (2) Periapical radiographs
Ultrasonography for noninvasive and real- time evaluation of peri-implant soft and hard tissue: a case series	Thöne-Mühling et al., 2021 [45]	Case series	3	Mindray ZS3 system with L30-8 high-frequency intraoral probe (resolution ~60 µm), B-mode imaging; standoff pad used in some cases for buccal scans	Post-operatively	Implants already placed and restored before the study (diagnostic evaluation only)	(1) Crestal bone level (implant threads) (2) Detection of buccal bone loss (3) Soft tissue thickness at peri-implant sites (4) Visualization of implant–abutment connection + surrounding peri-implant tissues	The ultrasound was used once to image implants that had already been in function for at least 6 years	(1) Intraoral radiographs (2) Intraoperative surgical findings
Ultrasonography for chairside evaluation of periodontal structures: A pilot study	Tattan et al., 2020 [46]	Prospective diagnostic pilot study	20	Prototype 24 MHz intraoral B-mode US probe with gel standoff pad (axial res. 64 µm, lateral 192 µm)	Pre-operatively	Single-tooth implant placement with flap reflection	(1) Interdental papilla height (2) Mid-facial soft tissue height (3) Mucosal thickness (tooth and edentulous ridge) (4) Soft tissue height at ridge (5) Crestal bone level	No follow up. The evaluations were all performed before the surgical procedure	(1) Direct periodontal probing (2) CBCT

## Data Availability

No new data were created or analyzed in this study. Data sharing is not applicable to this article.

## Supplementary Materials

The following supporting information can be downloaded at: https://www.mdpi.com/article/10.3390/jcm14228239/s1, File S1: Complete search strings and database-specific filters for all databases; File S2: List of Excluded Articles.

## Abbreviations

The following abbreviations are used in this manuscript:

US	Ultrasonography/Ultrasound Imaging
RCT	Randomized Controlled Trial
CBCT	Cone-beam computed tomography
OPG	Orthopantomogram
PRISMA	Preferred Reporting Items for Systematic Reviews and Meta-Analyses
Rob2	Risk of Bias 2
QUADAS-2	Quality Assessment of Diagnostic Accuracy Studies—version 2
JBI	Joanna Briggs Institute
HFUS	High-Frequency US
ICC	Intraclass Correlation Coefficients
CTG	Connective Tissue Graft
CAF	Coronally Advanced Flap
TUN	Tunnel Technique
STL	Standard Tessellation Language
CDV	Colour Doppler Velocity
PDI	Power Doppler Imaging
TKT	Thickness of keratinized tissue
CEJ	Cemento-enamel junction
MGJ	Mucogingival Junction
KM	Keratinized Mucosa width
MT	Mucosal Thickness
CBT	Crestal Bone Thickness
FBL	Facial Bone Level
BBD	Buccal bone dehiscence
STH	Supracrestal tissue height
STA	Soft tissue area
HSA	Hypoechoic supracrestal area
BRW	Bone ridge width
CBSQ	Crestal bone surface quality
GBR	Guided Bone Regeneration
FMT	Facial mucosal thickness
