# Supplementary material for: Diagnostic Applications of Ultrasound Imaging in Dental Implantology: A Systematic Review
Source: J Clin Med. 2025 Nov 20;14(22):8239. doi: 10.3390/jcm14228239 (PMC12653436; doi:10.3390/jcm14228239)
Supplement: Supplementary file 1 [file jcm-14-08239-s001.zip › SUPPLEMENTARY FILE S1.pdf]

Supplementary File S1: Complete search strings and database-specific filters for all databases

PUBMED

(("ultrasonography"[MeSH Terms] OR "diagnostic ultrasound"[All Fields] OR "ultrasound imaging"[All Fields] OR "intraoral ultrasound"[All Fields] OR "high-frequency ultrasound"[All Fields] OR "B-mode ultrasound"[All Fields])
AND
("dental implants"[MeSH Terms] OR "peri-implant tissue"[All Fields] OR "peri-implant mucosa"[All Fields] OR "peri-implant soft tissue"[All Fields])
AND
("diagnosis"[MeSH Terms] OR "imaging"[All Fields] OR "diagnostic imaging"[MeSH Terms]))
NOT
("therapeutic ultrasound"[All Fields] OR "ultrasound therapy"[All Fields])

SCOPUS

(TITLE-ABS-KEY("ultrasound" OR "ultrasonography" OR "ultrasound imaging" OR "intraoral ultrasound" OR "high-frequency ultrasound" OR "B-mode ultrasound"))
AND
(TITLE-ABS-KEY("dental implants" OR "peri-implant tissue" OR "peri-implant mucosa" OR "peri-implant soft tissue"))
AND
(TITLE-ABS-KEY("diagnostic imaging" OR "diagnosis"))
AND NOT
(TITLE-ABS-KEY("therapeutic ultrasound" OR "ultrasound therapy"))

WEB OF SCIENCE

TS=("ultrasound" OR "ultrasonography" OR "ultrasound imaging" OR "intraoral ultrasound" OR "high-frequency ultrasound" OR "B-mode ultrasound")
AND
TS=("dental implants" OR "peri-implant tissue" OR "peri-implant mucosa" OR "peri-implant soft tissue")
AND
TS=("diagnostic imaging" OR "diagnosis")
NOT
TS=("therapeutic ultrasound" OR "ultrasound therapy")

COCHRANE

(ultrasound OR ultrasonography OR "ultrasound imaging" OR "intraoral ultrasound" OR "high-frequency ultrasound" OR "B-mode ultrasound")
AND
("dental implant*" OR "peri-implant tissue*" OR "peri-implant mucosa" OR "peri-implant soft tissue*")
AND
("diagnosis" OR "diagnostic imaging")
NOT
("therapeutic ultrasound" OR "ultrasound therapy")
